# The future of phenomenological psychopathology

**DOI:** 10.1080/09515089.2024.2403881

**Published:** 2024-09-16

**Authors:** Lucienne Spencer, Matthew R. Broome, Giovanni Stanghellini

**Affiliations:** Department of Psychiatry, University of Oxford, Warneford Hospital, Oxford, UK; School of Psychology, Institute of Mental Health, University of Birmingham, Birmingham, UK; Birmingham Women’s and Children’s NHS Foundation Trust, Birmingham, UK; Department of Health Sciences, University of Florence, Italy; University “Diego Portales”, Santiago, Chile

The ways in which we attempt to make sense of psychiatric illness have been slow to progress. Over the last several decades, clinicians and researchers have inherited one-size-fits-all diagnostic frameworks (DSM, ICD) and limited interview techniques that have been slow to develop and adapt to their patients’ needs, intersectional identities and experiences. These modes of working have arguably been part of the lack of progress in mental health care and have resulted in less than satisfactory treatment success. In the search for alternative approaches to psychiatry and mental health care, there has been a reignited interest in phenomenological psychopathology: an approach that uses the phenomenological method to highlight the lived experience of the person with mental ill health and invites a person-centered approach to diagnosis and treatment. We believe phenomenological psychopathology may remedy many of the problems we currently encounter in psychiatric healthcare. The notion of a person-centered and more democratic approach to mental healthcare has a strong foothold in contemporary public discourse surrounding mental healthcare. While there is a continued risk of the objectification of patients, phenomenological psychopathology emphasizes their subjectivity and experience. The voice of the patient is first and foremost in our phenomenological understanding.

However, those who turn to phenomenological psychopathology as an answer to problems in psychiatric healthcare may find a philosophical tradition rooted in the early-mid 20th century that has done little to adapt to modern ideas in psychiatric healthcare and psychiatric research. The *Renewing Phenomenological Psychopathology* project, funded by the Wellcome Trust and led by Professor Matthew Broome and Professor Giovanni Stanghellini, calls for reflection, revitalization and reconstruction of this discipline, diversifying global scholarship and working with lived experience scholars, so that it can pave new paths in psychiatric understanding. The contributions of the current special issue aim to breathe new life into a vital method in psychopathology and to chart its future trajectory.

This introduction will take the following structure. It will begin with an overview of what phenomenological psychopathology is, both past and present. We will address why phenomenological psychopathology needs renewal, confronting its outdated features. Next, we will sketch an outline of some important ethical considerations to protect the rights of the patient within phenomenological psychopathology. We will then cover the contents of this special issue, which we have divided into two key themes: work that aims to apply phenomenological psychopathology in new ways and work that attempts to restructure the very foundations of the discipline. The aim of this special issue is not a severing of our roots. Rather, we hope to pull all that is fruitful in the tradition of phenomenological psychopathology into the present, opening it up to new possibilities.

## What is phenomenological psychopathology?

Advocates of the phenomenological method recognize that it is impossible to conduct an isolated investigation on the “mind” or “brain” of a psychiatric patient because embodied subjectivity is irreducible to a mere mind, and that we need a rich account of experience to understand what we are seeking to explain scientifically. Initially, the phenomenological approach, as exemplified by Jaspers (1912/1968), was influenced by work of the philosophers Franz Brentano and the early Edmund Husserl, with Jaspers fusing their approach with ideas from the hermeneutics of Dilthey and the sociology of Weber. This led to a “descriptive” phenomenological psychopathology, seeking to define and demarcate specific experiences present in mental illness, and their inter-relations. This approach opened a dual strategy for psychiatry, working in parallel, of empathic understanding and scientific explanation. However, the tradition subsequently took a more “transcendental” turn with Jaspers successors, notably in the work of Minkowski and Binswanger, whereby the field sought to infer the basic “structures” of experiences that unify and explain the more atomistic symptoms. These structures would include things like selfhood, affectivity, embodiment, interpersonal relations, and temporality. Hence, phenomenological psychopathology becomes a reflective account of the structures of experience of mental illness. For the phenomenological philosophers, such structures were thought to be universal or essential, whereas for their psychopathologist successors, where disorder and difference are key, there remains debate as to whether such structures are quasi-transcendental or indeed some would argue for there being key essential features of certain illnesses.

Although it is not wholly clear in their writings as to the methods employed by Minkowski, Binswanger and others, phenomenological psychopathology can surpass the limited scope of pre-structured interviews and diagnostic criteria by examining the patient’s lifeworld. And being open to new knowledge, rather than constrained by an a priori conception of the problems reified in semi-structured interview or questionnaire. After all, in the words of Stanghellini et al.: “we, as clinical psychiatrists, do not usually sit in front of a broken brain – we sit in front of a suffering person” (Stanghellini, 2019, p. 4). After a long period during which phenomenological psychopathology fell into obscurity, new work in the field and amplification of the patient’s voice through mad-pride activism and the role of those with lived experience have led to a resurgence of the approach, giving it a valued place amongst once more dominant methodologies.

## Why renew phenomenological psychopathology?

At this moment, phenomenological psychopathology is well-positioned to take a leading role in psychiatric care. And yet, as promising as phenomenological psychopathology might be in liberating our understanding of psychiatric illness, the tradition mustn’t fall prey to the same pitfalls encountered by alternative psychiatric approaches. As phenomenological psychopathology has done little to adapt its roots in the philosophy and social science of the early and mid-20th century, it risks falling into siloed thinking and, in turn, falling back into obscurity and irrelevance.

The founder of phenomenological psychopathology, Karl Jaspers, “passionately defended the need for methodological pluralism, emphasizing the extent to which methods and viewpoints from philosophy had a special value for psychiatry” (Zahavi & Loidolt, 2022, p. 58). Yet, more work needs to be done to imbue phenomenological psychopathology with contemporary viewpoints and methodologies, and the perspectives of those from diverse backgrounds.

A further concern is a lack of diversity in the field, not only on a disciplinary level but in virtue of the identities of the researchers themselves. Historically, phenomenological psychopathology has been dominated by white, European, male researchers without explicit lived experience of psychiatric illness. The very language that is used in this field may strike one as alienating and inaccessible. If the perspectives of these researchers are not challenged and expanded by those from more marginalized backgrounds, we continue to perpetuate a narrow and biased framework. What’s more, these groups who are more likely to face sexism, racism, homophobia, ageism, ableism and other stigmatization, are particularly vulnerable to poor mental health. A lack of inclusion in the field drives a hermeneutical injustice as certain under-represented voices are excluded from the interpretive framework (Fricker, 2007). Moreover, a lack of diversity in the field skews our understanding of psychopathology and inhibits the accuracy of treatment and diagnosis. Without challenges to our contemporary methodologies, aspects in our understanding of the mental health experienced by marginalized groups remain vacant.

It is for this reason that the *Renewing Phenomenological Psychopathology* project has constructed a wide research network of 54 members, which targeted those from non-WEIRD (western, educated, industrialized, rich, and democratic) countries. Our network spans across over 20 countries, including Singapore, India, Serbia, Romania, Japan, Egypt and South Africa. Moreover, our network includes those who are neurodiverse, those with lived experience of mental ill health and those with intersectional experiences. Thus, phenomenological psychopathology has been infused not only with new cultural insights but the insights of other underrepresented groups. In addition, we have funded translations for core texts in phenomenological psychopathology in Korean, Punjabi, Portuguese and Chinese. The International Exchange Awards provided by the Renewing Phenomenological Psychopathology project allowed researchers to travel across our networks and undertake a placement, in order to develop new interdisciplinary and cross-cultural insights into phenomenological psychopathology.

The project also created a unique co-production scheme. “Co-production” in mental health research acknowledges the valuable knowledge and expertise of people with lived experience of psychiatric illness or neurodiversity. It champions the production of joint research between experts by experience and academics/clinicians, who will contribute their insights equally:^1^

The contemporary scene … has witnessed a progressive uncoupling of academic psychiatry from front-line clinical care, an uncoupling that, corresponding with the two roles of phenomenology, presents both science-centered and individual- centered aspects … Its individual-centered aspect is reflected in the rise of “expertise-by-experience” standing alongside and in a co-productive relationship with traditional “expertise-by-training.” A key product of such co-production, unique to the contemporary period, is a model of “recovery” that is defined, not by the values of (by what is important or matters to) professionals as experts-by-training (such as diagnosis and symptom control), but by the values of (by what is important or matters to) patients and carers as experts-by-experience. (Messas et al., 2023, p. 12)

Through the co-production scheme, experts by experience were linked with one or more researchers from our international network, with the aim of co-producing a piece of work on the theme of renewing phenomenological psychopathology. This work involved a renewing of the methodology used in phenomenological psychopathology and a drawing out aspects of the lived experience of psychiatric illness that have previously been obscured.

Beyond the co-production of research, this scheme facilitated a mutual, two-way mentorship. All members of a collaborative team equally contributed with their knowledge and skillset (whether from the perspective of their expertise from experience or academic expertise) toward the production of research. Researchers and clinicians had the opportunity to gain valuable insight from experts by experience, and experts by experience developed their knowledge of research methodology, philosophy, phenomenology and academic practices. Through this scheme, we championed co-production in research and went some way toward amplifying the voices of people with lived experience in the field of phenomenological psychopathology.

By distributing research on phenomenological psychopathology beyond Western and disciplinary boundaries, we democratize knowledge to facilitate greater and more diverse input into the field. While we have made some headway toward diversifying this field, more work needs to be done. Our hope is that this special issue will inspire further projects in the diversification of phenomenological psychopathology.

## The responsibilities of phenomenological psychopathologists and the rights of patients

Since the nineteenth century, ethical principles and guidelines have been central to medical practice. While some ethical principles in medical practice overlap with psychiatry (consent, experimentation, confidentiality, prejudice, dignity, autonomy and so on), others are unique to psychiatric practice. Psychiatrists are required to make distinct ethical decisions, such as whether to use restraint on someone under the mental health legislation, whether to prescribe mind-altering medication and whether to disclose a stigmatized psychiatric diagnosis. There are also specific, vulnerable populations within psychiatric healthcare that may require unique ethical guidelines, such as those with intellectual disabilities, neurodivergence, children, older people and members of the LGBTQIA+ community. While there are epistemological goals in psychopathology, these ought not to be prioritized over the well-being of the patient. Indeed, our epistemological ambitions ought to be in line with improving the mental health of the patient and alleviating harmful symptoms.

In the course of renewing phenomenological psychopathology, it is essential that we rethink the ethical responsibilities of its practitioners. As a psychiatric approach that reinstates the subjectivity of the patient, phenomenological psychopathology ought to be even more sensitive to the vulnerabilities of patients and the responsibilities of clinicians compared to alternative approaches. However, there has largely been an absence of ethics from the theoretical framework in phenomenological psychopathology.^2^ Here, we outline some preliminary thoughts on the ethical responsibilities of those conducting phenomenological psychopathology and the rights of those seeking psychiatric help.^3^

Phenomenological psychopathologists ought to employ an attitude of modesty regarding their capacity to understand an experience they have not had.^4^ As a methodology that advocates for the first-person account of the phenomenology of a mental “illness”, it is one of the best therapeutic strategies we have for creating a rich account of the illness experience. However, only so much can be understood from a secondhand experience. In previous work, we have been critical of the phenomenon of empathic understanding being used as a direct (or semi-direct) insight into the experience of the patient (L. Spencer & Broome, 2023). Through hermeneutical collaboration with the patient, a complex and enlightening phenomenological account can indeed be pieced together; however, a complete understanding is not easy to attain and should not be assumed. For this reason, we advocate for a dyadic approach and for co-production with experts by experience in phenomenological psychopathology. By avoiding epistemic arrogance, we are likely to scrutinize the phenomenological accounts we produce rigorously and pay closer attention to the insights of the patient.

Under the umbrella of co-production, we encourage the approach of “co-writing” in phenomenological psychopathology: “Among the various forms of collaboration available in the literature … co-writing [is] a specific practice where a clinician and a patient are mutually engaged in jointly or collaboratively writing a narrative related to the patient’s experience” (Faccio et al., 2022, p. 123). As argued by Faccio et al, attention to personal stories and needs becomes particularly important in a historical and cultural background characterized by growing social and political pressures toward restoring old practices of social control and custodialism. This phenomenon has arisen an opposite pressure from other “subordinate” social agents who claim the right to challenge the dominant knowledge about mental illness and replace it with alternative knowledge, built not by mental health professionals but by the users themselves. In the recent past, the appearance on the scene of patient associations or patients’ families has only partially contributed to the progress of the clinical disciplines, leading instead sometimes to an acute and nonproductive conflict between the parties involved. All this makes it necessary to implement collaboration practices between professionals and users in view not only of the construction of effective treatment paths but also of the formulation of knowledge on mental discomfort and illness that arises from the dialogue between the various stakeholders and leads in the direction of a synthesis.^5^

This takes us to our next ethical responsibility: phenomenological psychopathologists ought to treat patients as invaluable epistemic agents. As a methodology that puts the testimony of the patient at the center of the phenomenological investigation, phenomenological psychopathologists are less likely to commit the testimonial injustices that have been identified in alternative approaches to psychiatric healthcare. Testimonial injustice occurs when a person’s testimony is not taken seriously by virtue of an identity prejudice (Fricker, 2007). Unfortunately, such testimonial injustice is common in psychiatric healthcare (Kidd et al., 2022; L. J. Spencer, 2021), and is a topic core to a further Wellcome Trust funded research project, Epistemic Injustice in Healthcare (EPIC).^6^ Phenomenological psychopathology, however, brings the voice of the patient to the fore, which directly informs their understanding of a given condition. We emphasize the importance of exercising reflexive, virtuous listening to the patient to protect their epistemic agency (L. Spencer & Broome, 2023).

So, too, phenomenological psychopathology attempts to throw out imposed interpretive frameworks, thus avoiding hermeneutical injustice. Hermeneutical injustice is another epistemic harm rife in psychiatric healthcare, which occurs when patients are excluded from the meaning-making process, leaving gaps where significant aspects of their experience ought to be (Fricker, 2007; L. J. Spencer, 2021) (Ritunnano, 2022) (L. J. Spencer, 2023). In contrast, at the heart of phenomenological psychopathology, from its founding in *General Psychopathology*, is a rejection of the interpretive structures that had come before (K. Jaspers, 1913/1997). The semi-structured interviews popular in phenomenological psychopathology (such as Interpretive Phenomenological Analysis, or the PHD interview method) continue this tradition of hermeneutical openness. Such epistemically just practices are essential for the autonomy and agency of the patient. However, we must not merely trade in the framework of the DSM for a phenomenological framework, expecting only the former to be restrictive and alienating. Rather, the patient ought to lead the meaning-making process, in their own words, to ensure hermeneutical justice.

Moreover, through a methodological plurality, we are more likely to avoid imposing stagnant interpretive frameworks that may miss critical experiential features. In recent research, Fernandez has explored the methodological compatibility between phenomenological psychopathology and qualitative research methods which can be used to strengthen our approaches overall (Fernandez, 2024). We propose further work into alternative means of expressing one’s phenomenological account beyond interview and written narrative, such as through artistic expressions. While this would likely need to be accompanied by verbal expression, alternative means of expression may capture some aspects of one’s experience that are particularly difficult to put into words. These distinct means of expression may be particularly suited to younger people or those with neurological difficulties such as dementia.

Finally, phenomenological psychopathology ought to avoid prejudice. Implicit biases are dangerously easy to commit and still permeate psychiatric healthcare (L. J. Spencer, 2021). Beyond adopting a reflexive awareness of implicit bias, it is also essential to adopt an intersectional attitude. Intersectionality has deep roots in Black feminist literature, championed by the likes of Audre Lorde (1977), bell hooks (1981) and Patricia Hill Collins (1990). However, this methodology is used to examine not only the intersections between sex and race but also other intersecting social identities such as disability, age, sexuality and psychiatric illness. Turning to the realm of psychiatry, Frantz Fanon (1952) argued that it was impossible to truly understand a Black person’s psychiatric illness outside their colonized situation. For Fanon, the colonized situation saturates every aspect of one’s existence. Consequently, he observes that the attempts of his fellow psychiatrists to examine a patient in abstraction from their colonized situation end in failure. Fanon is aware that one’s social situation permeates the meaning structures of one’s world. It gives one’s entire life-world a particular hue.

The work of Fanon, along with De Beauvoir, Sartre, Merleau-Ponty and other critical phenomenologists has gone on to inform “critical phenomenology” today. Critical phenomenology is an ethical and politically motivated framework that highlights the ways in which our social world affects our own intentional consciousness. Regardless of the popularity of critical phenomenology in other areas of the field, and despite its psychopathological roots in the work of Fanon, =, phenomenological psychopathology in its current form is insufficiently sensitive to the intersectional character of lived experiences. The patient’s life-world is structured by gender, race, ethnicity, age, sexuality and other characteristics that give their illness a unique meaning. Therefore, we should consider how these structural factors intersect at a primordial level of the illness experience. Ignoring these aspects of the patient’s identity gives us only a partial view of the patient’s life-world, thus obstructing psychiatric knowledge. To ignore the intersectional character of illness is to commit two key ethical harms: 1) it obscures our intellectual endeavors toward understanding a given condition; 2) it impedes the patient’s understanding of their illness, contributing toward a hermeneutical injustice and a lack of self-understanding.

We propose that further work ought to be done to develop an ethical framework that is interwoven into the very practice of phenomenological psychopathology.

## Phenomenological psychopathology applied to specific psychiatric conditions

This first part of the special issue will focus on how phenomenological psychopathology can be applied in new ways to gain a deeper understanding of specific psychiatric conditions. In the founding text of phenomenological psychopathology, *General Psychopathology*, Karl Jaspers applies the methodologies of the discipline to enrich our account of various experiences, including delusions, psychosis, dementia, hysteria and obsessions (K. Jaspers, 1913/1997). Indeed, one of the core achievements attributed to phenomenological psychopathology has been a richer understanding of an array of psychiatric experiences that had previously been limited to biological accounts. Yet, since 1913, not only has our understanding of these conditions drastically transformed, but entirely new conditions have been recognized and defined. Here, contributing authors will explore how phenomenological psychopathology can enrich our account of these conditions.

### Schizophrenia

As the title suggests, the focus of Pablo López-Silva’s paper “Thinking in Schizophrenia and the Social Phenomenology of Thought Insertion” is the experience of an external agent placing thoughts in one’s head; a typical symptom of schizophrenia. López-Silva challenges our understanding of thought insertion as lacking in agency from the individual with schizophrenia. This paper brings to the fore the social phenomenology of thought insertion and the multimodal nature of thinking in psychosis, thus challenging the standard approach to thought insertion. To conclude, López-Silva draws comparisons between the characterization of thought insertion and current research in social perception and clinical practice. Through this exploration, this paper develops a richer understanding of the phenomenology of schizophrenia.

In “Self-disorders in schizophrenia as disorders of transparency: an exploratory account”, Feyaerts, Nelson and Sass explore new approaches to the concept of the minimal self in the popular phenomenological account of schizophrenia. The authors identify two core problems that emerge from the phenomenological account of the minimal self in self-disorders. First, the minimal self cannot be both a universal feature of consciousness and a distinct feature of self-disorders in schizophrenia. Second, there is a conflict between there being an exaggerated “for-me-ness”, and there being a loss of self in schizophrenia (which is frequently reported). The authors propose that a “transparency view” might overcome some of the challenges of the minimal self-theory and may offer an alternative way of understanding the experiential abnormalities involved in self-disorders in schizophrenia.

For phenomenological psychopathology to be a meaningful, person-centered approach to psychiatric healthcare, Sofia Jeppsson argues that we need to ensure that the testimony of patients is being taken seriously. In “Allegedly Impossible Experiences”, Jeppsson highlights that while the aim of phenomenological psychology is to bring to light the experiences of those with mental ill health, certain experiences, namely those of delusions, are at risk of being dismissed as inconceivable. On the other end of the spectrum, some clinicians are overly confident in their capacities to understand complex delusional experiences, and draw premature conclusions about these experiences. Jeppsson proposes that phenomenological psychopathology ought to strike a balance between hasty dismissals and mistaken conceptions of delusion. In order to achieve this, it is essential that those with the given condition play a significant role in meaning-making. In this sense Jeppson proposes that methods of co-production are integrated into phenomenological psychopathology.

### Depression

In “Socialized into Depression – Towards A Social Phenomenological Psychopathology” Domonkos Sik draws on Merleau-Ponty’s phenomenology of ontology and applies an intersubjective framework to depression in order to determine the role of the “socialization” processes in the emergence of a depressed lifeworld. While there has been much work on depression as a form of “unworlding”, Sik characterizes depression as a disruption of “chiasm”: the synthesis of the bodily and the conscious, which are both rooted intersubjectivity (the relationship with the Other). In characterizing depression as a social pathology, the condition can be understood as a collapse in one’s intersubjective and intracorporal capacities. And yet, depression for Sik goes beyond a bodily, mental or even social suffering. There is an interrelated distortion of time consciousness, agency, and interaffectivity. Sik concludes by demonstrating the ways in which these sociological insights can impact therapeutic practice for people with depression.

In “Silence, depression, and bodily doubt: Toward a phenomenology of silence in psychopathology”, Dan Degerman uses the case-study of silence in depression in order to demonstrate what the phenomenology of silence can reveal about a given condition. Drawing on Merleau-Ponty, and previous work on this theme (L. J. Spencer, 2021), Degerman argues that silence acts as a backdrop to our being-in-the-world. Degerman develops the three basic kinds of silence experiences that emerges from Merleau-Ponty’s account – inner, outward, and outside silence. Silence is often an invisible experience, however it comes to the fore of our attention when there is a disruption in language. Degerman demonstrates that this is particularly the case in depression, giving rise to what Carel refers to as bodily doubt (Carel, 2016). Through a phenomenology of silence, we can develop a more rigorous account of the phenomenology of depression.

### Autism

While there has been burgeoning research on the phenomenological experience of Autism, there has been little work in this domain on the experience of camouflaging in Autism. In “Autistic trans camouflaging: an early phenomenological exploration”, Ruby Hake fills this gap by putting forward a phenomenological case study of the autistic trans experience of camouflaging. By delving into Simone de Beauvoir’s concept of “doubling”, Hake puts forward an account of camouflaging in autism as “doubling”, whereby one simultaneously experiences one’s authentic neurodiverse self and one’s projected neurotypical image. Hake goes on to argue that in the case of trans autism, there is a least a “tripling” through an experience of 1) the autistic self, 2) the born-body, 3) the neurotypical/right/cis-gender mask.

### Post traumatic stress disorder

Jake Dorothy and Emily Hughes enrich our current understanding of the phenomenology of Post Traumatic Stress Disorder (PTSD) in “The Death Of The Self In Posttraumatic Experience”. Drawing on testimonies from those suffering from PTSD, a fragmented self emerges: a self that is simultaneously alive and dead following a traumatic experience. The authors investigate this phenomenon further through Waldenfels’ conception of the split self. They find that the embodied conflict between having a body that is passively affected and being a body that actively understands takes on a heightened and debilitating state following trauma. The degree of the dislocation of the self surpasses what the lived body can handle. Consequently, through this split self, the person with PTSD experiences dissociation, indescribability, and the fragmentation and repetition of time. Dorothy and Hughes conclude by demonstrating how the notion of a split self can help inform therapeutic practice, as the authors encourage the pursuit of an intrapersonal bond with the part of the self that has been lost.

## Revitalising methodology

For the second edition of the special issue, these authors explore new means of revitalizing the methodology of phenomenological psychopathology. Although the vestiges of phenomenology can be found across disciplines, phenomenological psychopathology has done little to engage with fields outside of philosophy and psychiatry. Advances in disciplines such as anthropology, sociology, neuroscience, critical race theory, and linguistics offer exciting new opportunities which are missed by such a guarded approach. Through the Renewing Phenomenological Psychopathology project, we encourage an opening up to new disciplines by creating a network that spans various fields. In addition, we funded workshops and sandpit events to explore interdisciplinary connections in phenomenological psychopathology. The future of phenomenological psychopathology requires further heterogeneity. By forging constructive relationships between phenomenological psychopathology and alternative disciplines, we provide fertile ground for new insights into the experience of mental ill health and neurodiversity.^7^ In this second part of the special issue, we introduce phenomenological psychopathology to a variety of methodologies, including computational psychiatry, philosophy of education, virtue ethics and cognitive neurosciences.

### Redressing inequality

One vital pursuit of renewing phenomenological psychopathology is critically examining structures of power and inequality perpetuated by our traditional methodologies. In “Psychotherapy of The Oppressed: The Education of Paulo Freire in Dialogue with Phenomenology”, Piedade and Messas turn to the field of education in order to address injustices in the field. The authors compare the relationship between the therapist and the patient in contemporary approaches to psychopathology to that of the teacher and the student in the education system. Piedade and Messas introduce phenomenological psychopathology to the work of Brazilian educator Paulo Freire, who sought to adjust the oppressive power dynamics in the education system by redressing the teacher-student relationship. Through the backdrop of Freire and his pedagogical concepts, such as the “banking model of education” and the creation of “culture circles”, this paper seeks to find new ways of dispelling injustice in psychiatric healthcare. They call this contribution to the field a “Psychotherapy of the Oppressed”.

In “*Existential Injustice in Phenomenological Psychopathology*”, Vespermann targets a specific form of injustice that they identify as taking root in phenomenological psychopathology: that of affective injustice. Affective injustice occurs when a subject is wrongfully curtailed in their expression or experience of their feelings (Archer & Mills, 2019). The authors focus on a structural form of affective injustice, whereby one’s *distressing background feelings* are violated. Background feelings can be understood as “enduring feeling states that condition our perceptions of everyday situations, interpersonal dynamics, and the broader social milieu” (Vesperman, 2024: ?). They refer to this injustice of violating distressing background feelings as an *existential injustice*. Through the lens of existential injustice, the authors show how mental health conditions can evolve in deprived social contexts. This paper proposes that phenomenological psychopathology ought to be more sensitive to the social backdrop within which background feelings of distress are expressed.

In the last decade, there has been burgeoning philosophical research on what frameworks of intersectionality can offer to psychiatry. In “Radically Contextualising Mental Health: Interdisciplinary Contributions to a New Model for Tackling Social Differences and Inequalities in Mental Healthcare”, Baiasu and Messas contribute toward this literature by examining inequality in phenomenological psychopathology through the lens of intersectionality and the social relationality of the human situation. They echo a resounding cry to re-contextualize the subject of phenomenological psychopathology (Messas & Fernandez, 2022). The authors use case studies from the Brazilian context to anchor their paper and to illustrate how social factors (one’s race, gender, sexual orientation, etc) might intersect to produce unique experiences among different social groups (with a focus on vulnerable young people). The authors hope that this framework will influence not only the field of phenomenological psychopathology but clinical mental healthcare practices as a whole.

### Value & virtue

In response to recent collaborations between the two person-centered approaches, Alessandro Guardascione evaluates the compatibility of Stanghellini’s phenomenological-hermeneutic-dynamical (P.H.D) psychotherapy method with Fulford’s value-based practice. Following a comprehensive comparison between the theoretical, practical, and ethical underpinnings of the two approaches, in “Situating Evaluativism in Psychiatry: On the Axiological Dimension of Phenomenological Psychopathology and Fulford’s Value-Based Practice” Guardascione draws out some key distinctions in their conception of *value*. Patient values, or “needs”, play a significant role in diagnostic and therapeutic decision-making. According to Guardascione, the non-cognitivist and anti-realist position in Fulford’s values-based practice is incompatible with Stanghellini’s approach to phenomenological psychopathology, namely the normative claims on our affective and evaluative life. This paper calls for a consistent account of values across Phenomenological psychopathology and linguistic-analytic value-based practice for a meaningful and fruitful partnership to emerge.

Ensuring an ethically sound practice of phenomenological psychopathology requires reviewing not only the practice’s structures but also the specific conduct of phenomenological clinicians. In “Open-mindedness and Phenomenological Psychopathology: An intellectual virtue account of phenomenology and three educational recommendations”, Andrew Maile brings to the fore the epistemological effort clinicians in phenomenological psychopathology must employ, as psychopathology is essentially a form of psychiatric investigation and interpretation. Given this epistemological effort, Maile advocates for a pursuit of intellectual virtue in the training and education of clinicians who employ phenomenological psychopathology. Maile draws our three core aspects to such an intellectual virtue: good questioning, listening, and reflecting. This important paper contributes toward the urgent need for ethical practice in phenomenological psychopathology.

### New approaches to phenomenological psychopathology

Broeker and Broome seek to better understand the minimal self in schizophrenia in their paper “Minimal self” Locked into a Model: Exploring the Prospect of Formalizing Intentionality in Schizophrenia”. This paper challenges the descriptive and transcendental accounts in the phenomenology of schizophrenia. They then go on to apply the framework of Marr’s computational psychology to explore whether intentionality and the minimal self in the phenomenology of schizophrenia can be formalized into mathematical terms in a computer model, hence, examining potential routes to combine computational psychiatry with phenomenological psychopathology. Such an approach, takes phenomenology back to the concerns of Husserl in *The Logical Investigations* (Husserl, 1900/2001) and *Ideas 1* (Husserl, 1913/1970), where his interest in semantics and meaning utilized phenomenology to synthesize psychology and logic.

The special issue concludes with an exploration of clinical narrative in phenomenological psychopathology in “Clinical narrative and the painful side of conscious experience”. Ramírez-Bermúdez, González-Grandón and Chávez propose that to reinvigorate phenomenological psychopathology we need to take seriously clinical storytelling as a tool for recovering the meaning of mental symptoms. Such clinical narrative brings to light social, intersectional, cultural and personal significance in the psychiatric experience. This tradition amalgamates medical, neuroscientific, psychotherapeutic, and literary texts. Not only does this approach improve our understanding of a given condition, but it also increases the agency of psychiatric patients as their narrative is brought to the fore.

## Notes

1 We recognize, however, that clinicians and academics themselves may have lived experience of mental ill health, and those with lived experience may have existing clinical/academic skills.

2 Rosfort demonstrates that ethics is essential for meaning-making as it is an imperative aspect of human experience; therefore a phenomenological investigation is incomplete in the absence of ethics (Rosfort, 2019).

3 We often use the term patient but our arguments regarding “rights” applies to those seeking psychiatric help more broadly who may not be classed as patients.

4 Some phenomenological psychopathologists will have direct experience of the condition they are examining. This critique is targeted at the many who do not have this requisite lived experience.

5 For more on this, see: Faccio, E. Pocobello, R. Vitelli, R & Stanghellini, G. (2022) “Grounding co-writing: An analysis of the theoretical basis of a new approach in mental health care” https://doi.org/10.1111/jpm.12835:

6 For further information on the EPIC project see https://wellcome.org/grant-funding/people-and-projects/grants-awarded/epic-epistemic-injustice-health-care.

7 See Fernandez (2024) on interdisciplinary approaches to phenomenological psychopathology.
